# Ovarian endometrioid carcinoma with sex cord-like features: a case report with genomic and transcriptomic analyses and literature review

**DOI:** 10.3389/fonc.2026.1826424

**Published:** 2026-05-29

**Authors:** Shanshan Guo, Qianjue Tang, Jie Zhang, Wei Zhao, Siru Chen, Hong Xia, Chunyan Zhang, Xiaoyun Zhang

**Affiliations:** 1 Departments of Gynecology, Longhua Hospital, Shanghai University of Traditional Chinese Medicine, Shanghai, China; 2 Departments of Central Laboratory for Science and Technology, Longhua Hospital Shanghai University of Traditional Chinese Medicine, Shanghai, China; 3 Science and Technology Research Department, Yueyang Hospital of Integrated Traditional Chinese and Western Medicine, Shanghai University of Traditional Chinese Medicine, Shanghai, China; 4 Departments of Pathology, Longhua Hospital, Shanghai University of Traditional Chinese Medicine, Shanghai, China

**Keywords:** CTNNB1, morphological remodeling, sex cord-like ovarian endometrioid carcinoma, transcriptome sequencing, Wnt/β-catenin signaling pathway

## Abstract

Endometrioid carcinoma resembling sex cord-stromal tumour (SCLEC) is a rare histologic variant of ovarian endometrioid carcinoma characterized by trabecular, nested, or retiform growth patterns resembling sex cord-stromal tumors, while preserving the immunophenotypic and molecular features of endometrioid differentiation. Accurate diagnosis requires careful histopathologic assessment and exclusion of true sex cord-stromal neoplasms. We report a 71-year-old woman who underwent comprehensive staging surgery and was diagnosed with left-sided low-grade ovarian endometrioid carcinoma with sex cord-like features (stage IA1). Hematoxylin and eosin (HE) staining and immunohistochemistry(IHC) demonstrated morphologic resemblance to sex cord tumors, particularly granulosa cell tumors, with positivity for epithelial markers and negativity for sex cord differentiation markers. Molecular subtyping and transcriptomic sequencing identified a p.D32Y missense mutation in exon 3 of CTNNB1. Transcriptomic profiling showed that the WNT pathway was the dominant oncogenic driver, whereas the Rap1 pathway was significantly enriched but transcriptionally repressed. These findings suggest that disruption of adherens junctions and cell-cell adhesion may contribute to loss of epithelial polarity and the development of sex cord-like morphology. The patient remains in remission with no evidence of disease. To our knowledge, this is the first reported case of ovarian SCLEC characterized by transcriptome-based molecular profiling.

## 1 Introduction

Ovarian endometrioid carcinoma with sex cord-like structures (OEC-SCLS), also referred to endometrioid carcinoma resembling sex cord-stromal tumour (SCLEC), is a rare histological variant of ovarian endometrial cancer(OEC). It is characterized by morphologic features resembling sex cord-stromal tumors, including trabecular, nested, or retiform growth patterns, while retaining the immunophenotypic and molecular characteristics of endometrioid differentiation. Accurate diagnosis requires careful histopathologic evaluation and exclusion of true sex cord-stromal neoplasms. Some patients may present at an early stage with clinical features mimicking ovarian sex cord-stromal tumours. This tumor is generally considered to have low malignant potential and a prognosis similar to that of conventional OEC, with favorable outcomes typically observed in early-stage cases.

We report the case of a 71-year-old woman who presented with cyclical vaginal bleeding for six months, which may be associated with the molecular subtype of the tumor. Following comprehensive staging surgery, the lesion was evaluated by routine pathologic examination, molecular subtyping, and Transcriptomic Analyses. We also reviewed all previously reported cases of SCLEC to further explore the molecular mechanisms underlying its distinctive sex cord-like morphology. The patient is currently in remission and remains disease-free.

## 2 Case presentation

**Figure 1:**
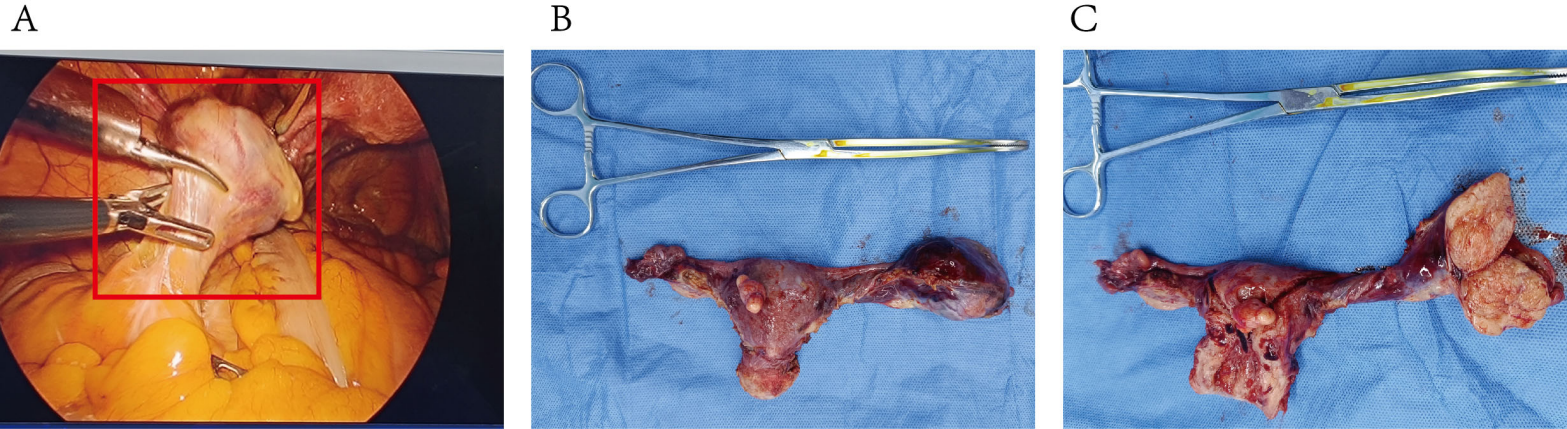
Gross and intraoperative findings. **(A)** Laparoscopic view of the left ovary. **(B)** Photograph of the postoperative specimen. **(C)** Cut surface of the left ovary.

A 71-year-old female presented in February 2025 with a six-month history of cyclical vaginal bleeding, occurring at intervals of approximately 25-35 days and with an estimated volume of about 20 mL per episode. Before presentation, she had undergone two hysteroscopic curettage procedures and one colposcopic biopsy. No significant abnormalities were identified during or after these procedures, and her symptoms persisted despite surgical intervention. On admission, contrast-enhanced pelvic MRI showed no obvious abnormalities. Biochemical testing revealed an elevated estradiol level(E2: 164 pmol/L), whereas other relevant parameters were within normal limits (FSH: 24.7 IU/L, LH: 16.5 IU/L, P: 3.4 nmol/L, AMH <0.01, CA125:5.9 U/mL, HE4: 66 pmol/L). As none of the preoperative examinations suggested malignancy, laparoscopic total hysterectomy with bilateral salpingo-oophorectomy was performed as a preventive treatment. Intraoperatively, the left ovary was found to be enlarged, measuring 4×3×3 cm, and a 3 cm uterine fibroid was also noted. No significant abnormalities were observed in the right ovary or in either fallopian tube (**Figures 1A, B**). Gross postoperative examination showed that the left ovary had a solid cut surface (**Figure 1C**).

### 2.1 Pathological examination and molecular testing

Gross examination: The uterus measured 70*30*25 mm and included the cervix with a visible external os. The uterine dimensions, including a corpus diameter of 20 mm, cervical canal length of 25 mm, uterine cavity depth of 44 mm, endometrial thickness of 5-19 mm, and myometrial thickness of 13-17 mm, were within normal limits according to published reference ranges. A gray-white nodule measuring 20×12×12 mm was identified in the anterior uterine wall. On cut section, the nodule was gray-white, firm, and showed a whorled appearance. The left fallopian tube measured 90 mm in length and 2-5 mm in external diameter, with the fimbrial end identifiable. Adjacent to it was a gray-white to gray-yellow mass measuring 40×30×20 mm. On cut section, the mass was gray-white to gray-yellow, firm, and well circumscribed. The right fallopian tube measured 55 mm in length and 3-5 mm in external diameter, with the fimbrial end identifiable. The right ovary measured 20×15×5 mm and showed a gray-white to gray-yellow, firm cut surface. The left ovarian tumor measured 40×30×20 mm and was diagnosed as low-grade ovarian endometrioid carcinoma resembling a sex cord tumor, with focal stromal luteinization. Based on the morphologic and immunohistochemical findings, the final diagnosis was left-sided low-grade ovarian endometrioid carcinoma with sex cord-like features (stage IA1). No tumor involvement was identified in the left fallopian tube, right adnexa, or uterus.

**Figure 2:**
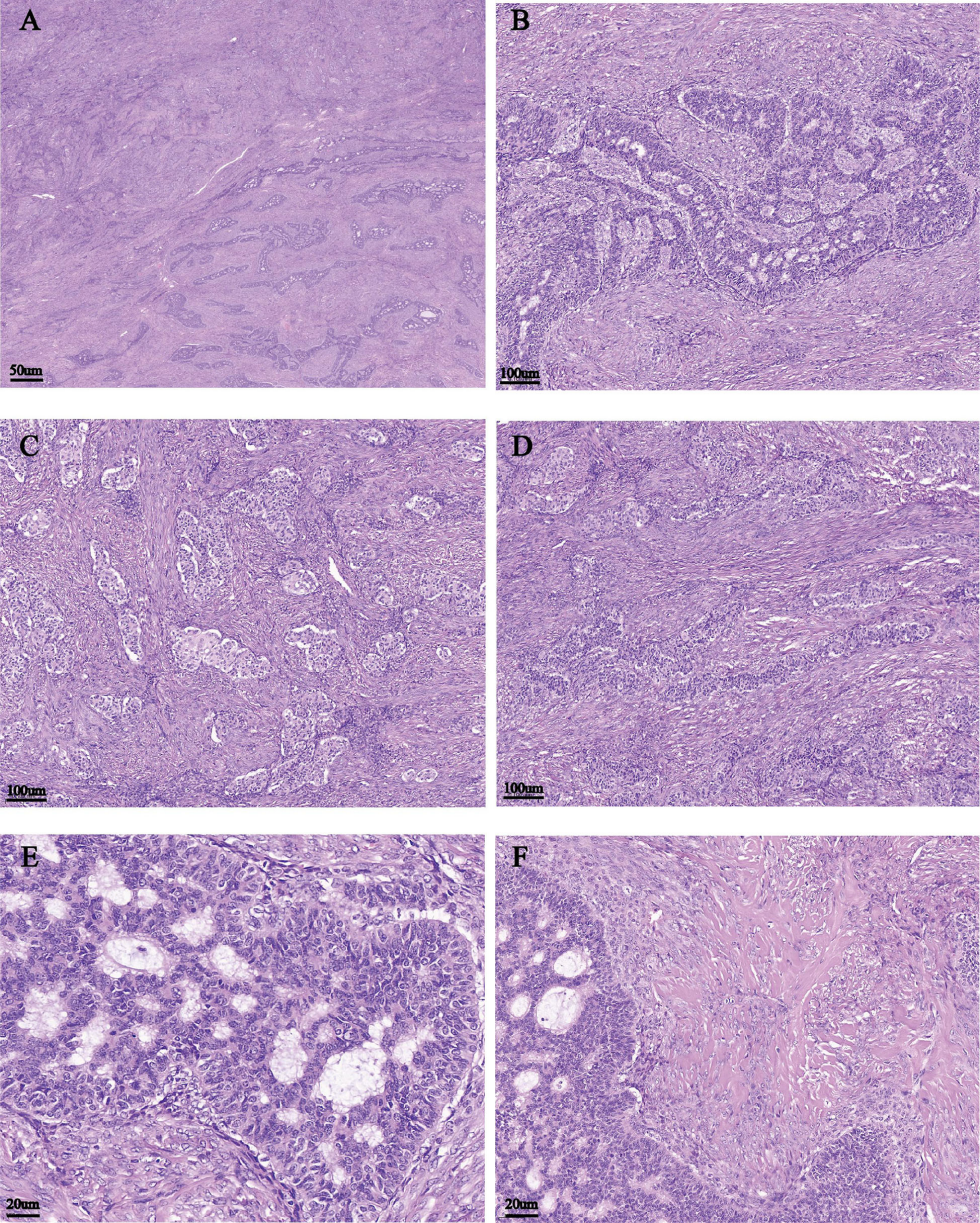
Histopathologic features of the tumor on hematoxylin and eosin (H&E) staining. **(A)** Tumor cells were embedded in fibrous stroma and showed diverse architectural patterns, including trabecular, cord-like, and small nested arrangements (upper left), as well as larger nests (lower right). Scale bar = 50μm. **(B)** Cribriform and follicle-like structures were observed within the large nests. Scale bar = 100μm. **(C)** Tumor cells arranged in solid small nests. Scale bar = 100μm. **(D)** Tumor cells arranged in solid trabecular and cord-like structures. Scale bar = 100μm. **(E)** Tumor cells within the large nests were round to ovoid and relatively bland, with scant cytoplasm and hyperchromatic nuclei. The nuclei showed irregular contours, thickened membranes, and visible nuclear grooves. These cells formed cribriform and follicle-like structures reminiscent of Call-Exner bodies in granulosa cell tumors. At the periphery of the nests, the tumor cells were round to ovoid with pale-staining nuclei, and the stroma showed hyaline degeneration. Scale bar = 20μm. **(F)** Marked stromal hyaline degeneration was observed in some areas. Scale bar = 20μm.

Microscopic observation (**Figure 2**): Histologically, the tumor was located within the ovarian parenchyma. The tumor cells were embedded in a fibrous stroma and arranged in trabecular, cord-like, and small- to large-nested patterns. Cribriform and follicle-like structures were observed within the larger nests (**Figures 2A–D**). The tumor cells in the large nests were round to oval and relatively bland in appearance, with scant cytoplasm, hyperchromatic nuclei, irregular nuclear contours, thickened nuclear membranes, and conspicuous nuclear grooves. These cells formed sieve-like and follicle-like structures, resembling granulosa cell tumors with Call-Exner body-like features. Cells surrounding the follicle-like spaces were round to oval with pale-staining nuclei, and the stroma showed hyaline degeneration (**Figure 2E**). In some areas, stromal hyalinization was particularly prominent (**Figure 2F**), no areas of endometrioid adenocarcinoma were seen.

**Figure 3:**
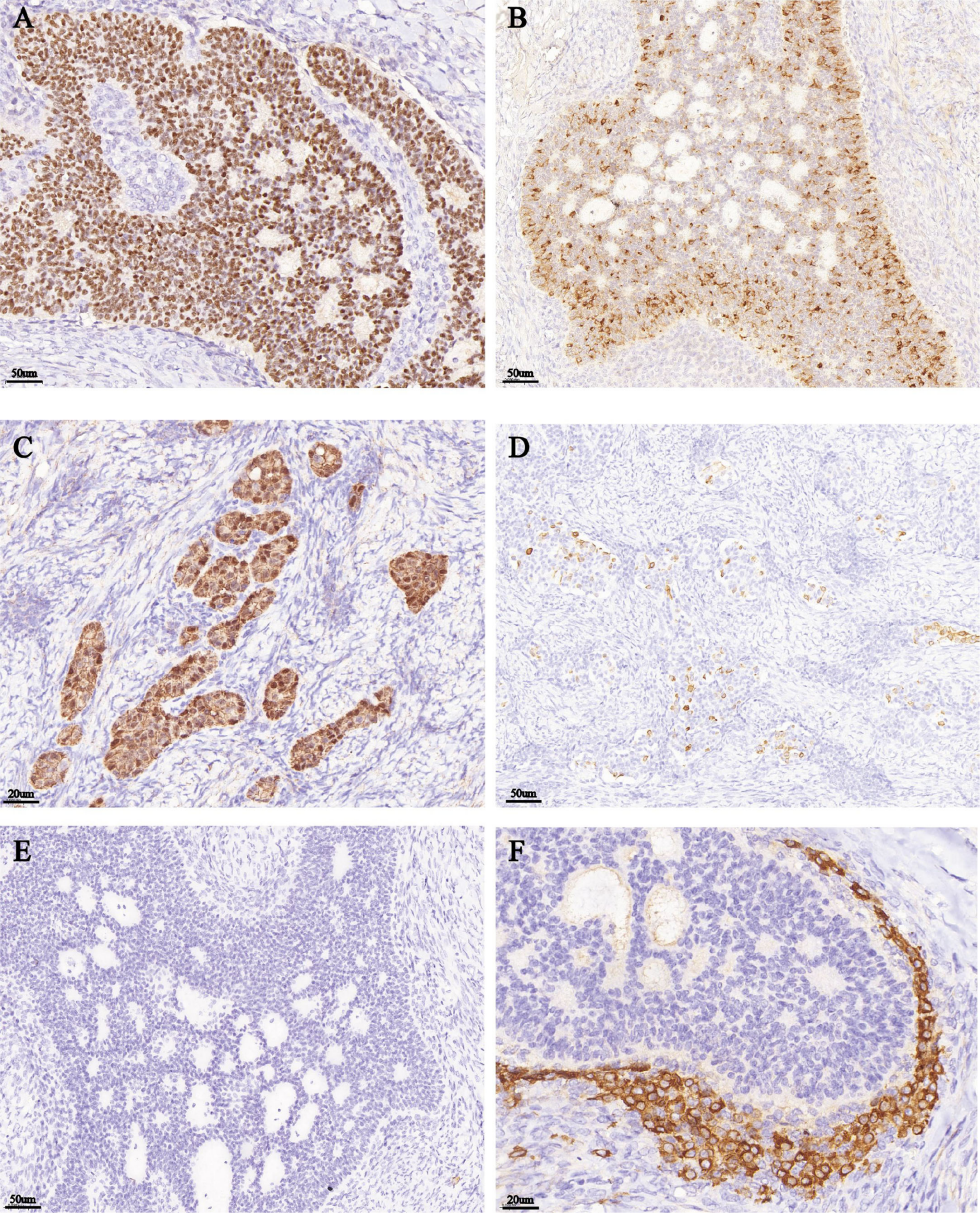
Immunohistochemical features of the tumor. **(A)** Tumor cells were positive for ER, whereas the perinest stromal cells were negative. Scale bar = 50μm. **(B)** Partial EMA positivity was observed in some tumor cells. Scale bar = 50μm. **(C)** β-catenin showed predominantly membranous staining with focal nuclear positivity. Scale bar = 20μm. **(D)** CK7 showed focal positivity. Scale bar = 50μm. **(E)** Tumor cells were negative for PAX8. Scale bar = 50μm. **(F)** Tumor cells were negative for α-inhibin, whereas the surrounding stromal cells were positive. Scale bar = 20μm.

Immunophenotype: The tumor cells were positive for AE1/AE3, ER (**Figure 3A**), PR, and CD10, with partial positivity for EMA (**Figure 3B**). β-catenin showed predominantly membranous staining with focal nuclear positivity (**Figure 3C**). CK7 was focally positive (**Figure 3D**). The Ki-67 labeling index was approximately 10%. The tumor cells were negative for PAX8 (**Figure 3E**), α-inhibin, SF-1, calretinin, FOXL2, and vimentin. Notably, the surrounding luteinized stromal cells were positive for α-inhibin (**Figure 3F**), SF-1, calretinin, and FOXL2, while remaining negative for the epithelial marker AE1/AE3.

Molecular analysis:

Tissue samples from the left ovarian tumor were selected for molecular subtyping analysis using a 27-gene endometrial carcinoma panel based on high-throughput sequencing with PCR-targeted capture technology. The panel assessed 27 genes associated with endometrial carcinoma, together with microsatellite instability (MSI) status. It included genes relevant to the molecular classification of endometrial carcinoma, such as exons 9-14 of the POLE gene, which encode the exonuclease domain, and the full coding sequence (CDS) of TP53; Lynch syndrome-related genes, including the mismatch repair genes MSH2, PMS2, MLH1, and MSH6; the CDS of EPCAM; as well as hotspot mutation sites and coding regions of 20 additional genes. The assay was designed to detect single-nucleotide variants (SNVs), small insertions/deletions (indels), and gene amplifications.

**Figure 4:**
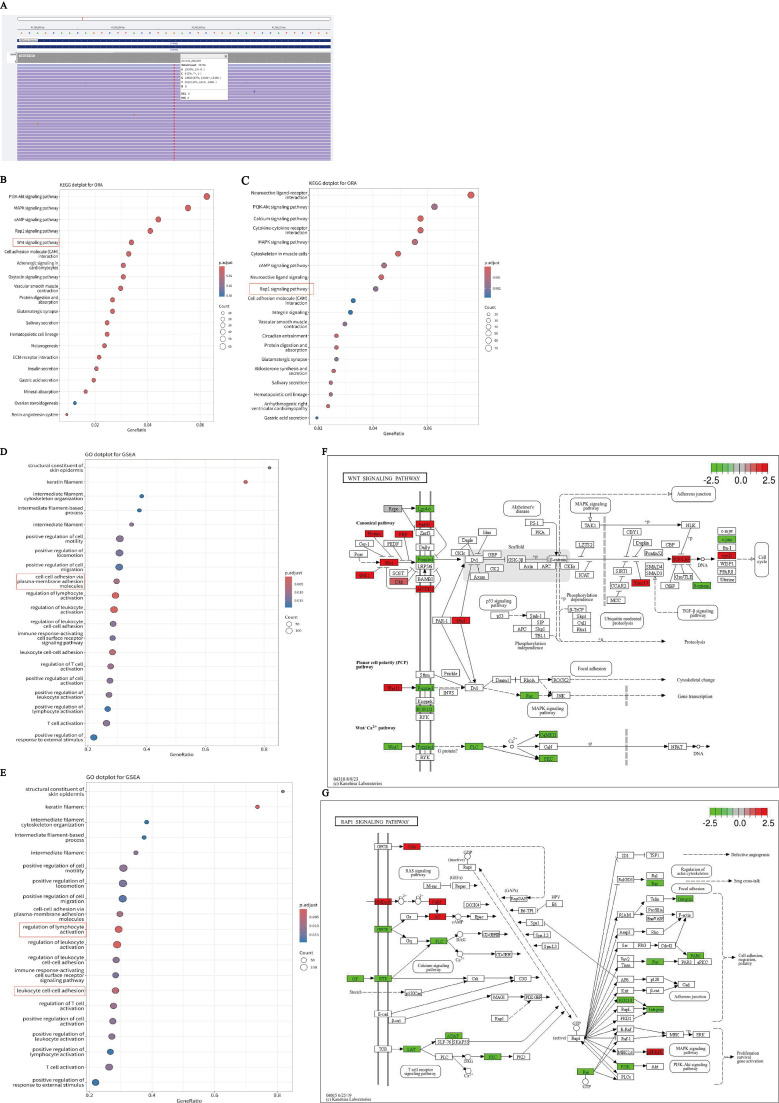
Molecular and transcriptomic findings. **(A)** Detection of a p.D32Y missense mutation in exon 3 of CTNNB1 by 27-gene molecular subtyping analysis for endometrial carcinoma. **(B)** KEGG pathway enrichment analysis showed significant enrichment of the WNT signaling pathway. **(C)** KEGG pathway enrichment analysis showed significant enrichment of the RAP1 signaling pathway. **(D)** Significant enrichment of the cell-cell adhesion pathway. **(E)** Gene set enrichment analysis (GSEA) showed significant downregulation of immune-related pathways, including regulation of lymphocyte activation and leukocyte cell-cell adhesion. **(F)** The canonical Wnt signaling pathway showed marked upregulation of the key LEF1/TCF transcription factor complex (red-highlighted node). **(G)** The Rap1 signaling axis and its downstream effectors showed marked transcriptional suppression (green nodes).

No pathogenic mutations were identified in POLE or TP53, and the tumor was microsatellite stable (MSS), consistent with a nonspecific molecular profile (NSMP). NSMP is the most common molecular subtype of endometrial carcinoma and is characterized by intermediate prognosis and substantial heterogeneity. In addition, a p.D32Y missense mutation in exon 3 of CTNNB1 was detected (**Figure 4A**), a variant previously associated with poor prognosis in endometrial carcinoma.

### 2.2 Transcriptomic analysis results

Given the rarity of SCLEC, its tumor heterogeneity and interactions with the microenvironment remain poorly understood. In this study, we applied transcriptomic sequencing for the first time to explore its underlying molecular mechanisms.Right ovarian tissue, used as normal control tissue, and left ovarian tumour tissue were selected for comparative transcriptome sequencing. The results showed significant enrichment of the WNT pathway (padj≈0.004), identifying it as the core driver pathway (**Figure 4B**) and highlighting its central role in canonical Wnt signaling. This pathway is characterized by upregulation of the LEF1/TCF complex, a key transcription regulator that initiates the expression of downstream target genes, as indicated by the red-highlighted node in **Figure 4F**. Among morphogenesis-related pathways, the RAP1 pathway, which is involved in cell adhesion and migration, was also significantly enriched(padj≈0.0018) (**Figure 4C**). In addition, the cell-cell adhesion pathway, which is essential for the formation of intercellular junctions, showed significant enrichment (**Figure 4D**). Notably, pathway mapping suggested that the Rap1 signaling axis and its downstream effectors were transcriptionally suppressed, as reflected by the green nodes (**Figure 4G**). Gene set enrichment analysis (GSEA) of the immune microenvironment further demonstrated significant negative enrichment of immune-related pathways, including regulation of lymphocyte activation and leukocyte cell-cell adhesion, indicating overall downregulation of immune-associated signaling (**Figure 4E**).

### 2.3 Literature review

To assess the prevalence and clinicopathologic characteristics of reported cases, we conducted a systematic literature search using bilingual keywords. The search strategy used “ovarian endometrioid carcinoma AND sex cord” in Chinese databases (Wanfang, CNKI, and VIP) and “(endometrioid carcinoma of the ovary) AND (sex cord)” in English databases (PubMed and Web of Science). This search identified 22 published studies reporting a total of 82 cases of SCLEC since its first description, as summarized in **Table 1**. The included studies comprised individuals aged between 22 and 89 years. CA125 levels were elevated in the majority of cases. Tumor size ranged from 0.6 to 43 cm. Among the cases with available staging information, 79.41% (27/34) were classified as FIGO stage I. Among cases with available follow-up data, the disease-specific mortality rate was 11.43% (4/35). Overall, most patients had a favorable prognosis, particularly those who underwent comprehensive staging surgery (30/35).The most common presenting symptoms included a palpable mass, abdominal pain, menstrual irregularities, and postmenopausal bleeding. Some patients also presented with vaginal bleeding, Morphologically, the majority of tumors exhibited a mixture of sex cord-like and endometrioid features. A minority (4/56) displayed purely sex cord-like morphology (including the present case).The primary tumor was unilateral in 77 cases and bilateral in 5 cases. In advanced stages, patients exhibited peritoneal or lymph node involvement (**Supplementary Material 1**).

**Table 1:** Reported cases of ovarian endometrioid carcinoma with sex cord-like features.

Authors	Cases	Age (years)#	CA125 (U/ml)	Diameter (cm)*	Molecular testing	FIGO	Intervention	Follow-up (months)#
Subbaiah (1)	1	65	67	15	NR	IIIC	Surgery + CT	Ongoing
Katoh (2)	4	60-79	NR	9-16	NR	IA, IC	Surgery/Surgery + CT	7-48
Fujibayashi (3)	1	52	24.2	8.5	NR	NR	Surgery	17
Wei (4)	1	39	82.2	11	NR	NR	Surgery + CT	24
Sookram (5)	1	72	159	22.7	Comprehensive genomic profiling	IA	Surgery	36
Travaglino (6)	9	50-89	NR	7-18.5	NGS	NR	NR	NR
Lengyel (7)	17	33-78	NR	0.6-27	NGS	IA to III	NR	NR
Talia (8)	1	30	723	10	NR	IC2	Surgery	NR
Gupta (9)	1	NR	NR	NR	NR	NR	Surgery	NR
Hu (10)	1	64	NR	15.5	NR	NR	NR	NR
Li (11)	1	70	191.4	11	NR	NR	Surgery	NR
Ma (12)	1	61	Negative	12	NR	NR	Surgery	NR
Young (13)	13	58-86	NR	4-43	NR	NR	NR	24-144
Yang (14)	1	77	Positive	8	NR	NR	Surgery	NR
Li (15)	1	32	NR	13	NR	NR	Surgery	Ongoing
Roth (16)	4	22-74	NR	11-30	NR	NR	Surgery	72-168
Matadial (17)	1	71	NR	5-16	NR	NR	NR	NR
Guerrieri (18)	6	55-83	NR	5-20	NR	IA to III	Surgery+RT/Surgery/Surgery+CT	1-68
Ordi (19)	13	60	NR	14.8	NR	NR	NR	10-120
Li (20)	1	71	NR	5	NR	NR	NR	6
Xu (21)	2	64-67	Positive	12-11	NR	NR	NR	Ongoing
RemadiS (22)	1	71	NR	25	NR	NR	Surgery	Ongoing
Our case	1	71	5.9	4	Transcriptomic Analyses and Molecular typing of 27 genes	IA1	Surgery	Ongoing

NR, not reported; CT, Chemotherapy; RT, Radiotherapy; NGS, DNA-based next-generation sequencing. *, Data are presented as the maximum diameter of the mass for individual cases, with ranges minimum–maximum provided for multiple cases. #, Multiple cases using the minimum-maximum range as a display.

### 2.4 Outcomes and follow-up

The patient underwent surgery without complications and had an uneventful postoperative recovery. Based on intraoperative findings and final pathological examination, she was diagnosed with left-sided low-grade ovarian endometrioid carcinoma with sex cord-like features (stage IA1). According to the clinical guidelines (23), patients with stage IA ovarian cancer may be managed with postoperative surveillance alone. The patient is currently disease-free during follow-up.

## 3 Discussion

The diagnosis of this case was particularly challenging because of its rarity and its close morphologic resemblance to granulosa cell tumor. Histologically, the tumor displayed a sex cord-like architecture without the conventional features of ovarian endometrioid carcinoma. In addition, PAX8 expression was absent, which is unusual for ovarian endometrioid carcinoma but has been reported in SCLEC (7),. Given the patient’s cyclic vaginal bleeding and elevated estrogen levels, granulosa cell tumor was a key consideration in the differential diagnosis. Nevertheless, the tumor showed epithelial differentiation, with positivity for EMA and CK7 and absence of sex cord markers, favoring endometrioid carcinoma over a true sex cord-stromal neoplasm. The identification of a CTNNB1 mutation further supported this interpretation.

The 27-gene molecular profiling and comparative transcriptomic sequencing performed with a reference control provide important insight into the molecular basis of this disease. Previous studies have shown that approximately 30% of primary ovarian endometrioid adenocarcinomas harbor CTNNB1 mutations (24), and in our literature review, CTNNB1 alterations were identified in most previously tested cases.CTNNB1 encodes β-catenin, a central mediator of canonical Wnt signaling. The p.D32Y mutation identified in this case affects exon 3, disrupts a critical phosphorylation site, and likely prevents β-catenin degradation, resulting in its cytoplasmic accumulation and nuclear translocation (25). In the nucleus, β-catenin interacts with TCF/LEF transcription factors and activates downstream targets such as CCND1, thereby promoting tumorigenesis (26). Although only limited nuclear β-catenin staining was observed immunohistochemically, transcriptomic analysis demonstrated marked upregulation of the LEF1/TCF complex and significant CCND1 expression, supporting activation of the Wnt/β-catenin signaling axis. These findings further support the role of CTNNB1 mutation as an important molecular driver in this tumor.

Furthermore, our transcriptomic analysis suggested transcriptional repression of the Rap1 signaling pathway, despite significant enrichment of the cell-cell adhesion pathway. Rap1 is a small GTPase that is essential for the regulation of intercellular adhesion and cytoskeletal organization. In its active GTP-bound state, Rap1 promotes the assembly and stabilization of adherens junction complexes at the plasma membrane, thereby preserving epithelial polarity (27, 28). Conversely, inhibition of Rap1 signaling (**Figure 4G**) may lead to disassembly of adherens junctions and impairment of stable cell-cell adhesion. Such changes could disrupt epithelial architecture and contribute to a morphologic shift from conventional glandular or tubular structures to a loose cord-like growth pattern. This mechanism may partly account for the distinctive sex cord-like morphology observed in this tumor.

**Figure 5:**
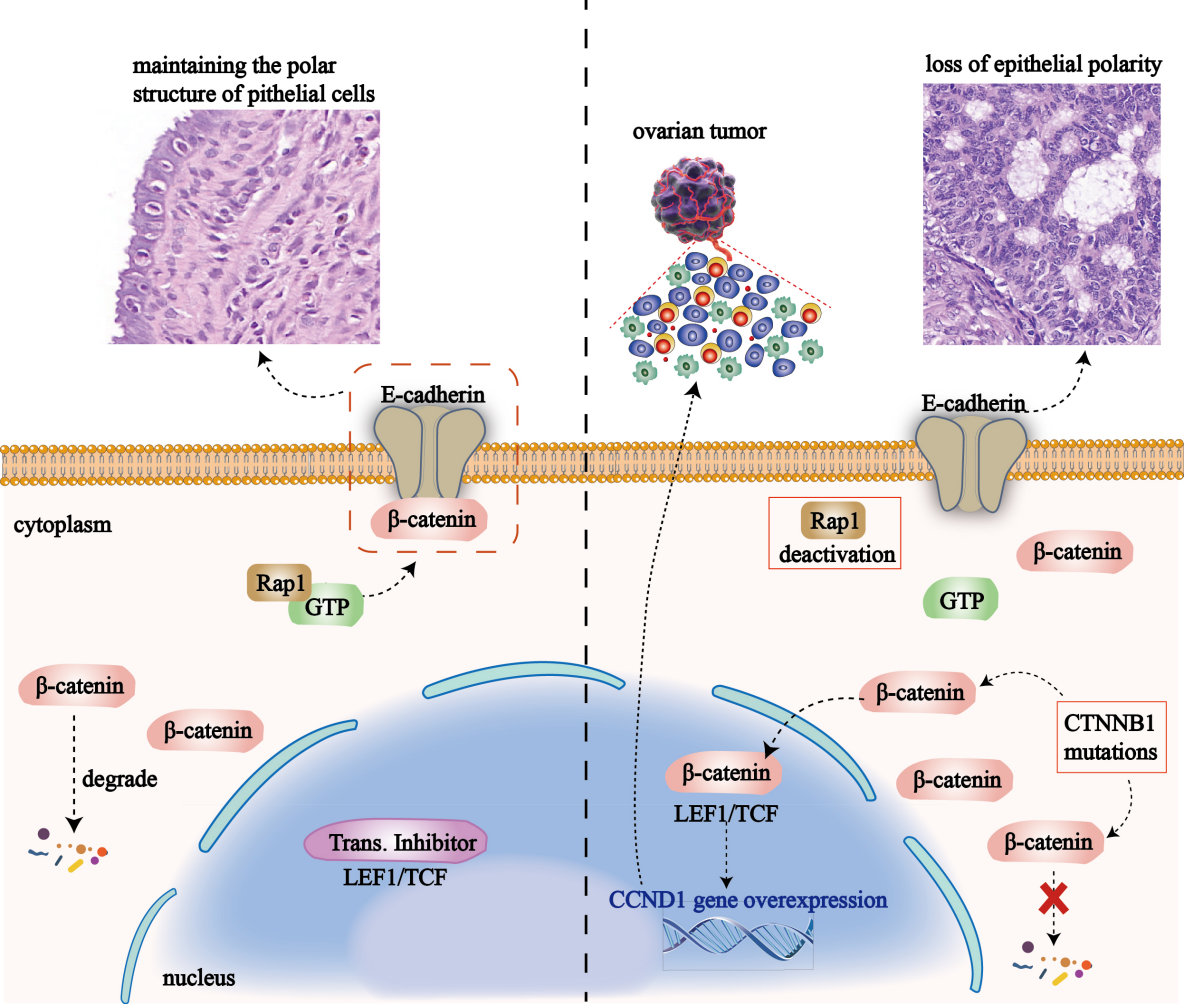
Proposed mechanism underlying SCLEC tumorigenesis and morphologic variation. Left panel: Maintenance of normal epithelial homeostasis. In normal epithelial cells, cytoplasmic β-catenin is tightly regulated and continuously degraded, thereby maintaining low nuclear β-catenin levels and preserving LEF/TCF in a transcriptionally repressive state. Active Rap1-GTP promotes stabilization of the E-cadherin/β-catenin complex at the plasma membrane, ensuring robust cell-cell adhesion and preservation of epithelial polarity. Right panel: Proposed pathological mechanisms in ovarian tumors. Mutation of CTNNB1 prevents normal degradation of β-catenin, resulting in its abnormal accumulation in the cytoplasm. Stabilized β-catenin then translocates to the nucleus, where it binds to LEF/TCF transcription factors and converts them from repressors to activators, thereby inducing overexpression of downstream target genes such as CCND1 (Cyclin D1), promoting uncontrolled cell cycle progression and tumor development. Meanwhile, inactivation of the Rap1 signaling pathway leads to loss of GTP-bound Rap1 activity, destabilization of the membrane-associated E-cadherin/β-catenin complex, and possible disruption of epithelial polarity, which may contribute to the development of sex cord-like morphology.

The hypotheses regarding CTNNB1 mutation-driven tumorigenesis and Rap1 pathway inhibition-induced morphological transformation are illustrated in **Figure 5**.

Available evidence suggests that activation of the Wnt/CTNNB1 signaling pathway is associated with a “cold” immune microenvironment. In CTNNB1-mutant endometrial cancer, negative enrichment of pathways related to lymphocyte activation and leukocyte adhesion supports the presence of an immune-excluded phenotype (29). Mechanistically, constitutive β-catenin activation may impair dendritic cell recruitment and reduce T-cell infiltration into the tumor microenvironment (30). Although these findings may have therapeutic implications, particularly in relation to immunotherapy response, further validation in larger cohorts is needed.

We also identified luteinized stromal cells expressing sex cord markers around the tumor cells. These stromal changes may explain the patient’s elevated postmenopausal estrogen levels and cyclical vaginal bleeding. Because CTNNB1 mutations have been described in both ovarian and endometrial carcinomas, it is possible that these tumors share overlapping molecular mechanisms (31–33). Whether they arise from a common origin, however, remains uncertain.

This study has several limitations. First, as a single-case analysis, its findings are inherently limited in generalizability, and the molecular observations should be considered preliminary and hypothesis-generating. Second, the absence of omics-level comparisons with conventional ovarian endometrioid carcinoma and pure sex cord-stromal tumors limits our ability to confirm the distinct biological basis of this subtype. Third, although our data suggest a possible association between CTNNB1 mutation and sex cord-like morphology, functional studies are still needed to establish a direct mechanistic link. Finally, because this tumor is extremely rare, the number of reported cases remains small, which may limit the strength of the statistical analyses.

## 4 Conclusions

Ovarian endometrioid carcinoma with sex cord-like features is a rare and diagnostically challenging tumor that requires integrated morphologic, immunophenotypic, and molecular evaluation. Transcriptomic analysis in this case revealed activation of the WNT pathway and suppression of the RAP1 pathway, providing preliminary insight into the molecular basis of its distinctive morphology. Published cases suggest that this tumor is usually detected at an early stage and is associated with a favorable prognosis. Our case further highlights that occult malignancy may be present despite normal imaging and tumor markers, supporting the clinical value of timely surgical management in selected patients.

## Data Availability

The datasets presented in this study can be found in online repositories. The names of the repository/repositories and accession number(s) can be found below: https://www.ncbi.nlm.nih.gov/, PRJNA1433694.
