# Supplementary material for: Ovarian endometrioid carcinoma with sex cord-like features: a case report with genomic and transcriptomic analyses and literature review
Source: Front Oncol. 2026 May 29;16:1826424. doi: 10.3389/fonc.2026.1826424 (PMC13259766; doi:10.3389/fonc.2026.1826424)
Supplement: Supplementary file 1 [file DataSheet1.doc]

TABLE 1 Study main characteristics.

| **Authors** | **Cases** | **initial symptoms** | **Laterality** | **Appearance** |
| --- | --- | --- | --- | --- |
| Subbaiah^[1]^ | 1 | Epigastric pain | Unilateral | SCL |
| Katoh^[2]^ | 4 | Atypical genital bleeding/abdominal distension/no symptom | Unilateral | SCL+EM |
| Fujibayashi^[3]^ | 1 | NR | Unilateral | SCL+ EM |
| Wei^[4]^ | 1 | Persistent dull lower abdominal pain | Unilateral | SCL+EM |
| Sookram^[5]^ | 1 | Progressive abdominal distention and pain | Unilateral | SCL |
| Travaglino^[6]^ | 9 | NR | Unilateral /Lymph node metastasis/Bilateral + peritoneal/Unilateral+peritoneal | SCL/SCL+EM |
| Lengyel^[7]^ | 17 | abdominal pain / abdominal distension/ Abdominal pain with weight gain and virilization | Unilateral /Bilateral | SCL/SCL+EM |
| Talia^[8]^ | 1 | abdominal pain | Unilateral | SCL+EM |
| Gupta^[9]^ | 1 | large pelvic mass | Unilateral | SCL+EM |
| Hu^[10]^ | 1 | abdominal distension | Unilateral | SCL+EM |
| Li^[11]^ | 1 | Right hip soreness and generalized fatigue | Unilateral | SCL+EM |
| Ma^[12]^ | 1 | Urinary frequency with lower abdominal distension and pain | Unilateral | SCL+EM |
| Young^[13]^ | 13 | NR | Unilateral/Bilateral | SCL/SCL+EM |
| Yang^[14]^ | 1 | Irregular vaginal bleeding | Unilateral | SCL+EM |
| Li^[15]^ | 1 | Bilateral swelling below knees | Unilateral | SCL+EM |
| Roth^[16]^ | 4 | left adnexal mass/postmenopausal  Bleeding/Painless ankle swelling/pelvic mass. | NR | SCL+EM |
| Matadial^[17]^ | 1 | NR | NR | NR |
| Guerrieri^[18]^ | 6 | NR | Unilateral/Bilateral | SCL/SCL+EM |
| Ordi^[19]^ | 13 | NR | NR | NR |
| Li^[20]^ | 1 | vaginal bleeding | Unilateral | SCL+EM |
| Xu^[21]^ | 2 | postmenopausal bleeding | Unilateral | SCL/SCL+EM |
| RemadiS^[22]^ | 1 | pelvic mass | Bilateral | SCL+EM |
| Our case | 1 | cyclical vaginal bleeding | Unilateral | SCL |

Abbreviations:NR, not reported;SCL, sex cord-like; EM, endometrioid.
